# Comparing methods for estimating leaf area index by multi-angular remote sensing in winter wheat

**DOI:** 10.1038/s41598-020-70951-w

**Published:** 2020-08-18

**Authors:** Li He, Xingxu Ren, Yangyang Wang, Beicheng Liu, Haiyan Zhang, Wandai Liu, Wei Feng, Tiancai Guo

**Affiliations:** 1 National Engineering Research Centre for Wheat, State Key Laboratory of Wheat and Maize Crop Science, Henan Agricultural University, Zhengzhou, 450002 People’s Republic of China; 2 Present Address: National Engineering Research Centre for Wheat, #62 Nongye Road, Zhengzhou, 450002 Henan People’s Republic of China

**Keywords:** Ecology, Physiology, Plant sciences

## Abstract

The reflectance of wheat’s canopy exhibits angular sensitivity, which can influence the accuracy of different methods for its leaf area index (LAI) estimation through multi-angular remote sensing. The primary objective of this study was to assess and compare the ability of various methods for LAI estimation from 13 view zenith angles (VZAs). The four methods included: (1) common hyper-spectral vegetation indices (VIs), (2) optimal two-band combination VIs (i.e., VIs: normalized difference index, simple ratio index, and difference vegetation index), (3) back-propagation neural network (BPNN), and (4) partial least squares regression (PLSR). Our results demonstrated that the red-edge plays a key role in estimating LAI, in that the traditional VIs, optimal two-band VIs, and PLSR including the red-edge band all showed satisfactory performance, with coefficient of determination (R^2^) > 0.72 in the nadir direction. However, the estimation accuracy of LAI was not positively related with band number, and BPNN gave unsatisfactory results under a larger viewing angle, with R^2^ ≤ 0.60 for extreme angles. The predictive ability of all four methods declined with an increasing VZA, with reliable LAI estimation near the nadir direction. Importantly, by comparing the four methods, PLSR emerged as superior in both its estimation accuracy and angular insensitivity, with R^2^ = 0.83 in the nadir direction and ≥ 0.65 for extreme angles. For this reason, we highly recommend it be used with multi-angular remote sensing data, especially in agricultural applications.

## Introduction

Leaf area index (LAI) is a key structural parameter and indicator of plants status. Since the LAI of wheat is a crucial morphological index for monitoring its current growth context and estimating its future yield, it forms an indispensable part of precision agriculture. Remote sensing is increasingly recognized as a reliable technique for LAI estimation, mainly because LAI has a strong relationship with spectral reflectance. Thus many studies have been carried out LAI estimation using remote sensing technique in recent years^1–4^.

Determining the LAI-sensitive band or band combination that most robustly estimates LAI from the vast and redundant hyperspectral information is a research “hotspot” in quantitative remote sensing. Early on, Guyot et al.^5^ showed that red-edge information is mainly influenced by plants’ structural parameters (i.e., LAI and leaf inclination angle) and pigment content; hence, the red-edge band or position could provide a useful tool for LAI estimation^4,6^. Common and widely used vegetation indices (VIs) were then developed and compared for their respective LAI estimation^7–9^. The canopy spectrum of plants is mostly influenced by their phenological period, leaf traits and environmental conditions, among other factors, leading to obvious regional characteristics and timeliness, thus precluding fixed optimal VIs across different regions. Furthermore, VIs with two or three bands are common remote sensing parameters, but they may be limited for exploiting the abundant information conveyed in narrow spectral bands of hyperspectral remote sensing data.

Many studies have used a non-parametric (i.e., neural networks) approach for monitoring crop LAI^2^, rice biophysical parameters^10^, maize nitrogen stress^11^, chlorophyll content^12^, and crop yield^13^. By comparing VIs and neural network methods for estimating LAI, Walthall et al.^14^ showed the scaled normalized difference vegetation index (NDVI) approach was the most effective method for LAI retrieval. In another investigation, after carrying out a continuum-removal analysis, the neural network method generally performed better than modified partial least squares^15^.

Alternatively, some researchers have concentrated on applying multivariate models for biophysical and biochemistry estimations. Partial least squares regression (PLSR) is full-spectrum technique that has been used to estimate biomass^16^, grassland LAI and chlorophyll^17^, rice nitrogen status^18^, and leaf water content^19^. When PLSR, principal component regression, and stepwise multiple linear regression were compared for assessing canopy pigment content in winter wheat, PLSR presented strong multicollinearity and insensitivity to sensor noise^20^. In a later study, Mirzaie et al.^21^ compared univariate and multivariate methods to estimate plant water content, finding that PLSR also provided the most accurate estimation ability.

All the above studies focused on the nadir direction. Canopy structure and soil background could affect the spectral data, which increases the difficulty of accurately identifying objects. The nadir direction mainly focuses on the information at the top of the canopy, while the contribution from the lower leaves of the plants is very small, so there is not enough information to extract the three-dimensional structure of the canopy. Meanwhile, analysis from the nadir direction seldom considers the influence of different proportions of soil background. But now, with the development of multi-angular remote sensing, directional information for describing canopy stereoscopic structure is accessible^22,23^. The proportion of soil background in the canopy can be lessened by adjusting the observation angle, and growth information on crops in the upper, middle and lowers layer can also be improved by using different observation angles. So, multi-angular remote sensing has been successfully used to estimate canopy LAI, forest biomass, and foliage clumping index^3,24,25^. In sum, compared with nadir direction observations, the retrieval of plant structural parameters using multi-angular hyperspectral remote sensing technique is a more powerful tool.

The relationships of VIs to LAI are known to change with the viewing zenith angle (VZA)^26^. Almost 20 years ago, Gemmell and McDonald^27^ argued that NDVI from off-nadir viewing angles should improve LAI estimation accuracy. However, work by Pocewicz et al.^28^ suggested viewing angles mattered little for the relationships between NDVI and LAI. Nonetheless, for shrubs, Stagakis et al.^29^ proposed their LAI estimation was possible with large VZAs. In particular, a “hotspot index” from multi-angular remote sensing could be used to improve LAI estimation than nadir’s NDVI^3,30^. Multi-angle studies do demonstrate that canopy structure information is more easily derived from non-vertical angles than a vertical angle of observation, which could thus reduce LAI estimation errors caused by crop structure characteristics.

Here, we examined the utility of different methods by applying hyperspectral measurements to estimate LAI under multi-angular remote sensing, including common hyperspectral VIs, optimal two-band combination VIs, back-propagation neural network (BPNN), and PLSR. We checked the angle sensitivity difference between the four methods, and analysed their respective influence of on monitoring capability. The coefficient of determination (R^2^) and root mean square error (RMSE) were used to compare and select the most suitable method for wheat. During 2011–2014, spectral recordings and LAI measurements were taken from the elongation through mid-filling stage of winter wheat that encompassed different cultivars, N treatments, and planting densities, which provided sufficiently high LAI variation for robust model development and testing.

## Results

### LAI estimation using traditional vegetation indices under 13 VZAs

**Figure 1:**
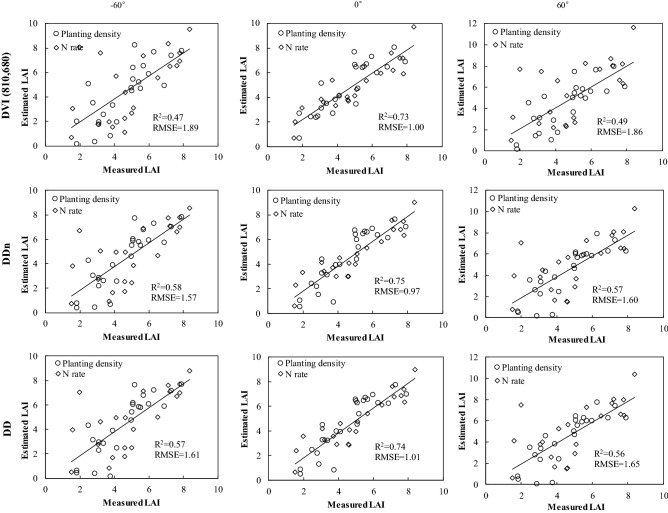
Measured vs. estimated LAI for DVI (810, 680), DDn, and DD indices under the nadir direction and extreme viewing angles.

We comprehensively compared the relationships between selected traditional 2-, 3- and 4- band VIs and LAI under different VZAs. As Fig. S1 shows, the R^2^ varied strongly with VZA, decreasing from 0° to extreme VZAs (± 60°) and reaching its peak value near 0°. Taking this nadir direction as reference, there was a slightly greater decline in R^2^-values for the forward than the backward scattering directions. The TCARI/OSAVI produced the largest difference between extreme angles, with 8.28% at − 60° versus 73.52% at + 60°, whereas EVI-1 had the smallest difference: 33.21% in − 60° viewing angle and 36.09% in + 60° viewing angle. Generally, the DVI (810, 680), DDn, mSR705, DD, and VOG-2 indices all showed good performance and were mainly composed of red-edge bands. Figure 1 shows the estimated and measured LAI at extreme viewing angles and nadir direction. In this respect, DVI (810, 680), DDn, and DD performed best among the 2-, 3-, and 4-band indices, respectively, with corresponding R^2^-values of 0.73–0.75 and RMSE-values of 0.97–1.01 in the nadir direction. However, these three models provided no advantage under extreme viewing angles (R^2^-values ≤ 0.58, RMSE-values ≥ 1.57). These results indicated that the ability of VIs to monitor LAI is affected by viewing angle.

### LAI estimation using normalized difference (ND), simple ratio (SR), and difference vegetation index (DVI) under 13 VZAs

**Figure 2:**
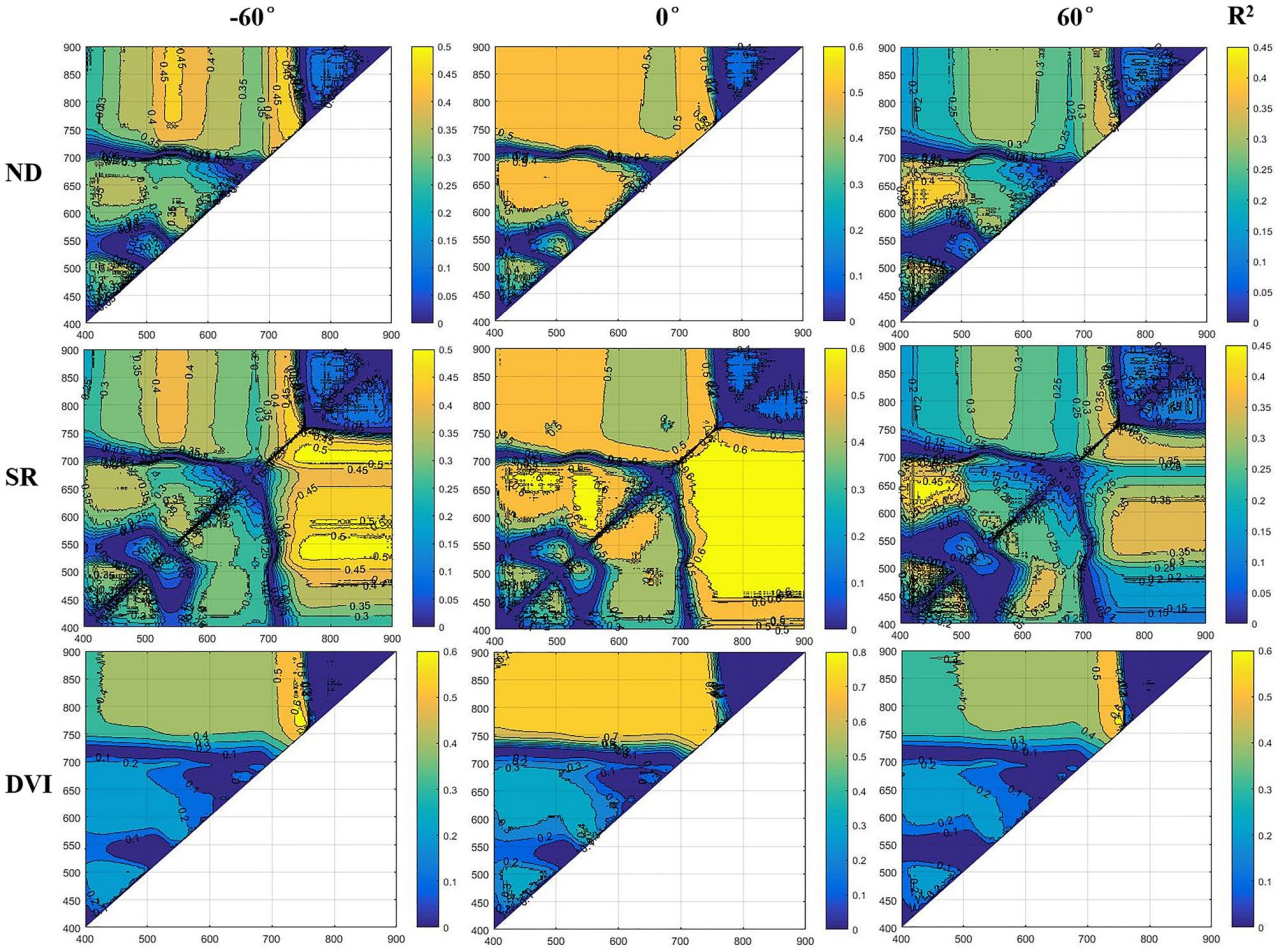
Contour maps showing the coefficient of determination (R^2^) for the relationships between the LAI and ND, SR, and DVI with extreme angles and nadir direction data formations.

Selecting optical spectral bands and bandwidths is an important consideration for any spectral index. Here, we compared any two random bands for an index (ND, SR, and DVI indices) in the 400–900 nm range to estimate LAI. In Fig. 2 is an example of such two-band combinations, from the nadir to extreme VZAs (± 60°), for which differences in spectral bands sensitivity among these three indices is obvious. For the ND and SR indices, sensitive bands for the forward directions are mainly in the blue-to-red band region, shifting to the red-edge band–red-edge band region for backward directions. For DVI indices, sensitive bands for all VZAs focused on the red-edge–red-edge region. These results also revealed several predictable trends: ND, SR, and DVI predictably decreased with increasing VZAs; the area of the sensitive region decreased with increasing VZAs, thus showing that the bands of the reduced region were sensitive to VZAs; the reserved band width, both in the nadir direction and extreme VZAs, was little influenced by VZAs.

**Figure 3:**
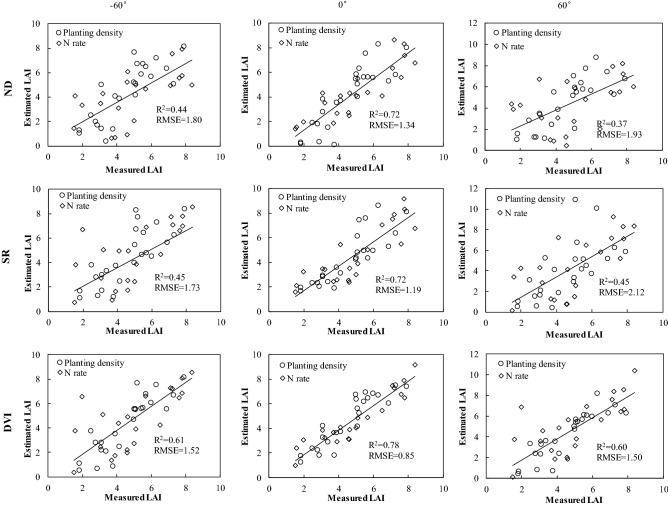
Measured versus estimated LAI for ND, SR, and DVI under the nadir direction and extreme viewing angles.

The optimized band position and R^2^-values for ND, SR, and DVI in 13 VZAs are listed in Table 1. The ND (748, 759), SR (770, 684) and DVI (736, 759) under the nadir direction had somewhat higher correlations with LAI than did larger VZAs. DVI (736, 759) showed the best performance in these three indices (R^2^ = 0.78). To predict LAI, linear regression models from extreme angles and nadir direction using optimal indices and LAI were applied (Fig. 3). The greatest accuracies were found for the DVI (736, 759) model under the nadir direction (R^2^ = 0.78 and RMSE = 0.85), which also outperformed both ND and SR under extreme angles.

**Table 1:** Selected sensitive wavebands for ND, SR, and DVI combinations using two separate wavelengths (λ_1_ and λ_2_), which had rather high R^2^ with LAI (leaf area index) at different VZAs (view zenith angles).

VZA	ND			SR			DVI		
	λ1	λ2	R^2^	λ1	λ2	R^2^	λ1	λ2	R^2^
− 60°	745	750	0.50	771	704	0.52	752	756	0.61
− 50°	745	750	0.53	771	699	0.57	752	756	0.63
− 40°	745	759	0.57	771	699	0.61	748	759	0.67
− 30°	745	759	0.60	771	688	0.63	748	759	**0.70**
− 20°	745	759	0.62	771	689	0.64	740	759	**0.73**
− 10°	745	759	0.62	771	689	0.65	741	759	**0.76**
0°	748	759	0.63	770	684	0.65	736	759	**0.78**
10°	468	648	0.60	487	648	0.65	736	759	**0.75**
20°	477	648	0.59	462	648	0.63	741	759	**0.72**
30°	487	648	0.57	462	648	0.61	748	759	0.68
40°	469	648	0.54	469	648	0.58	748	759	0.65
50°	434	640	0.50	468	649	0.54	752	771	0.62
60°	438	625	0.46	438	649	0.50	752	768	0.60

Numbers in bold represent results for *R*^2^ ≥ 0*.*70.

*ND* normalized difference, *SR* simple ratio, *DVI* difference vegetation index.

### LAI estimation using back-propagation neural network (BPNN) under 13 VZAs

**Figure 4:**
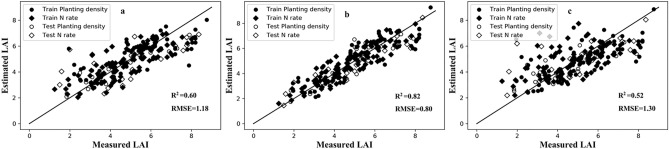
Measured versus estimated LAI for BPNN under the nadir direction and extreme viewing angles. (**a**) − 60°, (**b**) 0°, (**c**) + 60°.

When the BPNN was run under the 13 VZAs, its performance was similar to traditional VIs (Fig. S2). A comparative analysis of BPNN resulting from three representative VZAs is presented in Fig. 4. Evidently, since the prediction performance of BPNN depended on VZAs, with the latter increasing the R^2^ decreased. Its most accurate estimation of LAI was obtained at the nadir (R^2^ = 0.82, RMSE = 0.80), yet the back-scattering direction did show slightly higher accuracy than the forward direction. Hence, the monitoring accuracy of extreme angles was not greatly improved, with R^2^-values ≤ 0.60, RMSE-values ≥ 1.18.

### LAI estimation using PLSR under 13 VZAs

**Figure 5:**
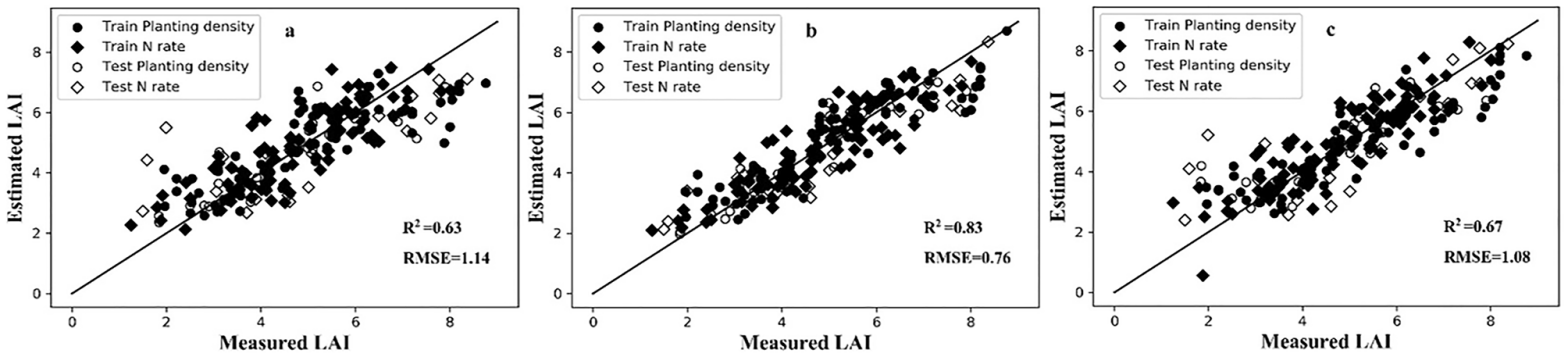
Measured versus estimated LAI for PLSR under nadir direction and extreme viewing angles. (**a**) − 60°, (**b**) 0°, (**c**) + 60°.

For PLSR, the optimal number of latent variables usable for LAI estimation can be defined through an analysis of RMSE values of the 13 VZAs (Fig. S3). This optimum ranged from 4 to 8; with 8 or more factors the RMSE increased rapidly, and at greater VZAs the RMSE also increased. Each band’s contribution to the PLSR latent variables can be expressed by factor loadings, as shown Fig. S4 for those values above the wavebands from 13 VZAs. The strong co-variation between LAI and reflectance in the red-edge regions can be discerned from positive peaks in the R^2^-values. High loading values were observed at waveband > 760 nm in the first latent variable, peaking at 715 nm for second variable and at 737 nm for the third variable. The loading weight value of the third latent variable is the highest, and the first latent variable is the lowest. Clearly, the selected narrow bands for the optimal DVI and PLSR showed strong agreement; in other words, the same spectral bands were critical in both methods. The estimation accuracy of LAI values was reduced as the VZA increased (Fig. S5). Compared with the VIs and BPNN, R^2^ of PLSR improved 10.7%, 6.4% and 1.2% than DDn, DVI and BPNN in the nadir direction, respectively; improved 17.5%, 11.7% and 28.8% than DDn, DVI and BPNN at a + 60° viewing angle, respectively; and improved 8.6%, 3.3% and 5.0% than DDn, DVI and BPNN at a − 60° viewing angle, respectively. Nonetheless, PLSR provide the best accuracy in the nadir direction (R^2^ = 0.83, RMSE = 0.76), with the monitoring accuracy of extreme angles greatly improved (R^2^-values ≥ 0.65, RMSE-values ≤ 1.11 (Fig. 5).

## Discussion

We have attempted to assess the performance of different methods for multi-angular remote sensing LAI data in this study. In comparing regression parameters derived from these four methods, we found that the effects of band positioning and band numbers were largely related to LAI-monitoring accuracy.

When estimating LAI, it is very important to select optimum bands or regions. Red-edge bands^4,31^, similar to the red-edge parameter and red-edge shape^1,32^, is strongly linked to LAI and strongly influenced by LAI. Consistent with this, all the methods investigated in our study confirm that the red-edge band corresponds most to LAI. The DDn, representing the traditional VIs, is composed of three red-edge bands (710, 660, and 760 nm) and was the best performer for monitoring LAI. Other VIs that include red-edge bands also showed good performance, such as the VOG and CI red-edge-1 (2-bands), mSR705 and MTCI (3-bands), and VOG-2, red-edge position and DD (4-bands). Yet sensitive red-edge regions were also detected for ND and SR in the backscattering direction, and for DVI under the 13 VZAs. The selected narrow bands in Table 2 and the noted high R^2^ values in Fig. 4 together confirm the red-edge is a crucial spectral region, one harbouring abundant information about LAI in wheat. However, this was not the case for the ND and SR in the forward scattering direction, which focused on the blue-red region. This emphasizes that traditional VIs are not suitable for all viewing angles, so we need to explore using sensitive VIs for multi-angular remote sensing. Fortunately, the high loading values for PLSR still rested on the red-edge band under 13 VZAs. These three methods—traditional VIs, optimal two-band VIs and PLSR—underscore the paramount important of red-edge bands for estimating LAI.

**Table 2:** Summary of the experimental design used for the collection of field measurements in this study.

	Time and site	Cultivar	Treatment	Sampling stages
N rate treatment (kg ha^−1^)	2011–2012	Zhengmai 366	0, 120, 240, 360 (Exp. 1)	Booting-initial-filling
	Zhengzhou	Yumai 49-198		
	2012–2013	Yumai 49-198	0, 120, 240, 360 (Exp. 3)	Jointing-initial-filling
	Zhengzhou	Zhengmai 9694		
	2013–2014	Yumai 49-198	0, 120, 240, 360 (Exp. 5)	Jointing-mid-filling
	Zhengzhou	Zhengmai 9694		
	2013–2014	Zhoumai 27	0, 180, 240, 300 (Exp. 7)	Booting-mid-filling
	Zhoukou			
Planting densities treatment (plants m^−2^)	2011–2012	Yumai 49-198	90, 180, 270, 360 (Exp. 2)	Booting-initial-filling
	Zhengzhou	Zhengmai 9694		
	2012–2013	Yumai 49-198	45, 90, 270, 360 (Exp. 4)	Jointing-initial-filling
	Zhengzhou	Zhengmai 9694		
	2013–2014	Yumai 49-198	45, 90, 270, 360 (Exp. 6)	Jointing-mid-filling
	Zhengzhou	Zhengmai 9694		
	2013–2014	Zhongmai 1	225, 450 (Exp. 8)	Booting-mid-filling
	Zhoukou	Aikang58		

Yet band number also proved valuable for LAI estimation in wheat, as different band numbers contain differing spectral information. Our results disagree with the view that more bands provide higher monitoring accuracy. Simple narrow-band indices method could serve as good predictors of LAI, especially when using the difference type VI. Our results further proved that a reasonable and possible estimation of LAI could be acquired from simple traditional VIs. Some, such as DVI (810, 680), DDn, and DD that represent 2 bands, 3 bands, and 4 bands indices, still achieved respectable predictive ability for LAI under the nadir direction.

Existing airborne sensors or satellite channels rarely employ accurately the same spectral bands as those identified and used in this study. Therefore, our work not only revealed optimum bands for the three type indices under 13 VZAs, but it also pointed to broad spectral regions highly correlated with LAI. These highlighted regions—blue band–red band region and red-edge band–red-edge band region—especially dominated the high R^2^ region under extreme viewing angles and are positively associated with LAI, which is promising for augmenting the use of current airborne sensors and satellite channels. As reported by others, VIs composed of blue band–red band and two red-edge bands are strong correlated with pigment content of vegetation^29,33,34^. In a previous study of ours, we demonstrated positive associations between both the blue band–red band region and two red-edge bands with leaf nitrogen content of winter wheat^35^. In the current study, we found the optimum DVI performed better than ND and SR under the 13 VZAs, which is consistent among traditional difference type VIs (DVI [810, 680], DDn, and DD). The advantage to an optimum DVI is that the sensitive region focuses entirely on the red-edge under the 13 VZAs, thus reducing the band setting for multi-angular sensors.

For the sake of contrast, BPNN was also tested in this study. Compared to the VIs methods, BPNN fed with full bands improved monitoring accuracy under the nadir direction, but also invited all spectral data into the model, which considerably increased its noise, run time, and complexity. Since its superiority was lacking, we may tentatively conclude that the estimation accuracy of LAI is not positively related with number of bands used. We acknowledge the BPNN method remains promising, especially if combined with another method, such as factor analysis and continuum-removal analysis, to improve its monitoring accuracy^15,36^.

Compared with the other investigated methods, the PLSR selects a subset of spectral bands related to LAI and removes unrelated information, which is at powerful advantage. In contrast to narrow-banded VIs, the PLSR is advantaged by its multivariate calibration; for example, similar to us, Hansen and Schjoerring^16^ also reported the PLSR analysis performed better than NDVI for estimating winter wheat biomass. Meanwhile, the noise of PLSR was relatively smaller than that of BPNN, and the first few factors of PLSR were more closely related to LAI than those of BPNN. This explains the more accurate results we got through PLSR over BPNN.

The sensor-viewing angle is an external factor when estimating crop vegetation properties by multi-angular remote sensing. In fact, canopy reflectance could change with the viewing angle, so the directional characteristics of surface reflectance should not be ignored^37^, hence, VIs, BPNN, and PLSR are further influenced by directional reflectance. Although sensor-viewing angle conditions may be calculated by different algorithms, such information is not normally used.

Many studies have focused on analysing angle sensitivity of VIs, reporting directionality effects arising from canopy structure, the proportion of photosynthetic tissues, shadow effects, and background compounds^38^. In our study, BPNN and PLSR consisted of multiple bands, for which angular sensitivity persisted, mainly because LAI was obtained from the whole plant leaves in the target area, and the larger viewing angles (i.e., ± 40°– ± 60°) mainly corresponded to the middle-upper part of plant properties of wheat^39^. Thus, inconsistency of spectral information and sampling probably led to the differential monitoring accuracy found under the 13 VZAs.

The angle sensitivity of the four compared methods, however, achieved their best estimation accuracy near the nadir direction. For BPNN, forward scattering direction did not showed better performance than the VIs, likely due to full bands diminishing the advantage of red-edge band. The performance disparity between backscattering and forward scattering directions for the spectral index and PLSR is small; perhaps because the red-edge band changes little with viewing angle, tempering this difference to some extent^35^. PLSR outperformed the other methods at larger viewing angles not only via its higher estimation accuracy but also its lower RMSE. This is best explained by the PLSR method selecting abundant related bands with LAI, and these bands were mainly focused on the red-edge bands. This suggests PLSR is suitable for multi-angular remote sensing.

The performance of red band, red-edge and NIR are different dependent upon VZAs; the red edge is relatively insensitive^35^. Some studies have used the sensitivity of the red and NIR bands to construct a hotspot index, which indeed improved the monitoring accuracy of LAI^3,30^. However, in actual production, the hotspot of reflectance varies according to the experimental date, location and measurement time, making the analysis of hotspots complicated. Thus, the application of hotspot index is limited. In this study, based on wide viewing adaptability as a starting point, by comparing four methods, we not only proved the importance of red edge monitoring LAI, but also demonstrated the insensitivity of the red edge to VZA. Thus, the red edge band could be an effective band when multi-angular remote sensing monitoring LAI on a large scale.

To date, multivariate calibration methods are used sparingly within agricultural remote sensing. So our findings here represent an initial step towards evaluating bilinear PLSR compared with VIs and BPNN. Although we tried to strengthen our dataset by including in it a wide range of LAI, soil types, cultivars and years, it cannot fully capture all the variability characterizing reality. To better increase the applicability of our results, more species and more satellite remote sensing data sets should be measured and tested. In addition, some radiative transfer models quantitatively describe the transmission mechanism of solar radiation in crop canopy, and fully consider the optical characteristics based on strict physical process and mathematics, which have strong universality and extensibility for data inversion. So the results of this study about multi-angular remote sensing should be tested and optimized using radiative transfer models in next step, and further analyze the application value of multi-angular data in agricultural production from the perspective of combining physical mechanism with biological mechanism. Nevertheless, our study was able to yield interesting insights concerning the usefulness of multi-angular remote sensing for estimating LAI in crops.

## Conclusion

Selecting suitable methods is necessary for multi-angular hyperspectral data analysis. This paper examined the most widely used VIs, optimal two-band VIs, BPNN, and PLSR models for LAI estimation under different VZAs. The results demonstrated that either of these methods is potential for establishing relationship between LAI and canopy reflectance under the nadir direction. Red-edge clearly plays a key role in estimating LAI, as the best traditional VIs, optimal two-band VIs, and PLSR all included a red-edge band in showing satisfactory performance. Portable sensors and satellite channels could thus pay more attention to selecting red-edge bands for monitoring other plants. Nonetheless, the predictive ability of the four methods decreased with increasing VZA, with LAI estimated most reliably near the nadir. Among all the methods, PLSR is superior to other in its estimation accuracy and angular insensitivity. This study increased angular application range for PLSR, revealing it as a powerful tool for multi-angular satellite remote sensing.

## Materials and methods

### Experimental data collection

The study area consisted of eight experiments from two experimental sites: Henan Agricultural University in Zhengzhou city (113° 35′ E, 34° 51′ N) and Shangshui Farm in Zhoukou City (114° 37′ E, 33° 33′ N). Overall, this represented two soil type (sandy loam and clay soils); two treatments (N rates and planting densities); two canopy structures: (erect and horizontal wheat cultivars); three consecutive years of data (2011–2014); and multiple growth stages of wheat (from jointing to mid-filling stages).

Each field experiment was arranged in a randomized complete block design, with three replications. The row spacing was 20 cm, and 50% of the nitrogen fertilizer (urea) was applied before each sowing stage, and the other 50% at the jointing stage for the + N treatments; P_2_O_5_ (as monocalcium phosphate [Ca(H_2_PO_4_)_2_]) at 150 kg ha^−1^ and K_2_O (as KCl) at 90 kg ha^−1^ were applied as base fertilizer for all treatments. The plant density was fixed at 1.8 × 10^6^ plants ha^−1^ for all N treatments; conversely, N applications were kept at 240 kg ha^−1^ for the planting density treatments, all of which corresponding to local farmers’ practice. Further details of this data collection are summarized in Table 2 and He et al.^35^. Since experimental factors will differentially affect canopy spectral reflectance, we collected a total of 221 plant samples to ensure adequate variation present in the possible range of LAI values acquired within each wheat cultivar, by including multiple growth periods, N and density treatments, years, and sites.

### Multi-angular spectral measurements and LAI measurements

Multi-angular spectral data were measured in situ by an ASD FieldSpec HandHeld spectroradiometer fitted with a 25°-field-of-view fiber optic adaptor and Field Goniometer System with a 1-m radius and view zenith angular resolution of 10° (Fig. S6). Data for 13 VZAs were obtained from backscattering extreme VZAs to the nadir direction to the forward scattering extreme VZAs (− 60° to 0° to + 60°). Back- and forward-scattering directions were designated negative and positive, respectively. The wavelength range used was 325–1,075 nm, and the sampling interval was the 3.5-nm band (Analytical Spectral Devices, Boulder, USA). For each VZA, ten sequential spectral reflectance values were obtained and then averaged for the analysis. To weaken the influence of solar observation angle on multi-angular data, all experimental data were acquired between 11:00 and 13:00, during which the solar zenith angle and azimuth angle varied little (Table S1).

Destructive LAI samples were taken at the same position where the multi-angular spectral data measurements had been taken. A leaf area meter (Model LI-3100, LI-COR, Inc., Lincoln, USA) was used to quantify total LAI from this harvested wheat material. For each experimental treatment plot, its mean LAI was based on five individual measurements of LAI that, overall, ranged between 1.25 and 8.76 (mean = 4.90, standard deviation = 1.63).

### Data analysis

#### Common hyper-spectral vegetation index

VIs represent a simple empirical approach for parameter inversion; hence, they could be widely used in forest, grass, and crop monitoring. Many VIs have been developed to estimate LAI, and those classed with 2, 3, or 4 bands are listed in Table 3.

**Table 3:** Common vegetation indices (VIs) used for leaf area index (LAI) estimation reported in the literature.

Index	Formular	References
**2 bands**		
Normalized Difference Vegetation Index [NDVI (810, 680)]	(R810 − R680)/(R810 + R680)	Rouse et al. (1974)^40^
Ratio Vegetation Index [RVI (810, 680)]	R810/R680	Jordan (1969)^41^
Difference Vegetation Index [DVI (810, 680)]	R810 − R680	Jordan (1969)^41^
Soil Adjusted Vegetation Index (OSAVI)	(1 + 0.16)(R800 − R670)/(R800 + R670 + 0.16)	Rondeaux et al. (1996)^42^
Photochemical Reflectance Index (PRI)	(R570 − R531)/(R570 + R531)	Gamon et al. (1992)^43^
Wide Dynamic Range Vegetation Index (WDRVI)	(0.2 × R800 − R670)/(0.2 × R800 + R670) + (1–0.2)/(1 + 0.2)	Peng et al. (2011)^44^
Vogelmann Index (VOG)	R740/R720	Vogelmann et al. (1993)^34^
Red edge Chlorophyll index (CIred-edge)	(R_NIR_/Rred-edge) − 1	Gitelson et al. (2003)^45^
**3 bands**		
Modified Red-edge Normalized Difference Vegetation Index (mND705)	(R750 − R705)/(R750 + R705 − 2 × R445)	Sims and Gamon (2002)^46^
Modified Red-edge Simple Ratio Index (mSR705)	(R750 − R445)/(R705 − R445)	Sims and Gamon (2002)^46^
Enhanced Vegetation Index (EVI-1)	2.5 × (R860 − R645)/(1 + R860 + 6 × R645 − 7.5 × R470)	Huete et al. (2002)^47^
Modified Chlorophyll Absorption in Reflectance Index (MCARI2)	[1.5 × (2.5 × (R800 − R670) − 1.3 × (R800 − R550))]/[Sqrt((2 × R800 + 1)^2^ − 6 × R800 + 5 × sqrtR670) − 0.5]	Haboudane et al. (2004)^8^
Modified Trangular Vegetation Index (MTVI2)	1.5 × [1.2 × (R800 − R550) − 2.5 × (R670 − R550)]/[(2 × R800 + 1)^2^ − (6 × R800 − 5 × R670^0.5^) − 0.5]^0.5^	Haboudane et al. (2004)^8^
Structure Insensitive Pigment Index (SIPI)	(R800 − R445)/(R800 − R680)	Peñuelas et al. (1995)^33^
Meris Terrestrial Chlorophyll Index (MTCI)	(R_NIR_ − Rred-edge)/(Rred-edge − Rred)	Dash and Curran (2004)^48^
New Double Difference Index (DDn)	2 × R710 − R660 − R760	LeMaire et al. (2008)^9^
**4 bands**		
TCARI/OSAVI (TCARI/OSAVI)	TCARI/OSAVI	Haboudane et al. (2002)^49^
Modified Soil-Adjusted Vegetation Index (MSAVI2)	0.5 × (2 × (R(760 − 900) + 1) − sqrt((2 × R(760 − 900) + 1) × (2 × R(760 − 900) + 1) − 8 × (R(760 − 900) − R(630 − 690)))	Qi et al. (1994)^50^
Red-edge Position	R700 + 40 × [(R670 + R780)/2 − R700]/(R740 − R700)]	Guyot et al. (1988)^51^
Double Difference Index (DD)	(R749 − R720) − (R701 − R672)	LeMaire et al. (2004)^52^
Vogelmann Index (VOG-2)	(R734 − R747)/(R715 + R726)	Vogelmann et al. (1993)^34^

#### Two-band random combinations

Two-band combinations indices were developed using two random available wavebands (i.e., λ1 and λ2) in the 400–900 nm range. This was done for three types of indices: a normalized difference (ND), simple ratio (SR), and difference vegetation index (DVI). As shown below in Eqs. (1)–(3):

1 $$ {\text{ND}}\;({\text{R}}_{\uplambda 1} ,{\text{R}}_{\uplambda 2} ) = \frac{{{\text{R}}_{\uplambda 1} - {\text{R}}_{\uplambda 2} }}{{{\text{R}}_{\uplambda 1} + {\text{R}}_{\uplambda 2} }} $$

2 $$ {\text{SR}}\left( {{\text{R}}_{\uplambda 1} ,{\text{R}}_{\uplambda 2} } \right) = \frac{{{\text{R}}_{\uplambda 1} }}{{{\text{R}}_{\uplambda 2} }} $$

3 $$ {\text{DVI}}\left( {{\text{R}}_{\uplambda 1} ,{\text{R}}_{\uplambda 2} } \right) = {\text{R}}_{\uplambda 1} - {\text{R}}_{\uplambda 2} $$

#### Back-propagation neural network

The BPNN is typically composed of input layers, some hidden layers, and output layers. As inputs, we used the 501 bands in the spectral range 400–900 nm; for the middle layer, the numbers of neurons were determined by the GridSearch^53^ to between 5 and 200; the activation function and optimizer of the middle hidden layer was “ReLU” and “Adam”. The fivefold cross-validation^54^ technique was used to model LAI in Python programming; the whole procedure was repeated 1,000 times to weaken unacceptable effects resulting from the random initialization of the optimization routine. For each VZA, 176 and 45 samples were used respectively as the training and test sets. The network outputs and the measured LAI values were used to build a linear regression model to identify the best BPNN model.

#### PLSR

PLSR was used to decompose the spectral data, by selecting several sensitive variables and remove some unrelated information to target the parameters. Reflectance data of 400–900 nm wavelengths were used for this PLSR analysis, which used the same training and test sets as BPNN. The GridSearch method was used to select the optimal number of PLSR factors, followed by a fivefold cross validation to assess model and select the best parameters.

#### Model performance

The spectral information of each VZA was processed and analysed separately as an individual set. First, the 221 samples were randomly partitioned into two databases: 176 samples for the calibration (training) set and 45 samples for the validation (test) set. Validation of the models for estimating LAI was done by comparing their R^2^ and RMSE values. For BPNN and PLSR methods, this relied on the fivefold cross-validation procedure, in which all samples are predicted once and only once. For the VIs’ methods, their corresponding R^2^ and RMSE values were calculated using Eqs. (4, 5).

4 $$ {\text{R}}^{2} = 1 - SSE{/}SST $$

5 $$ {\text{RMSE}} = \sqrt {\frac{1}{n} \times \sum\limits_{i = 1}^{n} {\left( {P_{i} - O_{i} } \right)^{2} } } $$

where *SSE* is the sum of squares for error, *SST* is the sum of squares for total, *n* is the number of samples, and *P*_*i*_ and *O*_*i*_ respectively are the predicted and observed values. The flow chart of experimental setup is given in Fig. S7.

## Supplementary information

Supplementary information.
