# Supplementary material for: Comparing methods for estimating leaf area index by multi-angular remote sensing in winter wheat
Source: Sci Rep. 2020 Aug 18;10:13943. doi: 10.1038/s41598-020-70951-w (PMC7435181; doi:10.1038/s41598-020-70951-w)
Supplement: Supplementary file 1 — Supplementary information. [file 41598_2020_70951_MOESM1_ESM.pdf]

Comparing methods for estimating leaf area index by multi-angular remote sensing in winter wheat

Li He, Xingxu Ren, Yangyang Wang, Beicheng Liu, Haiyan Zhang, Wandai Liu, Wei Feng*, Tiancai Guo*

Table and Figure legends

Table S1. Zenith and azimuth angles of the Sun for all study sites.

Fig. S1 The difference in performance between the VIs with LAI from 13 viewing angles. (a) 2 bands, (b) 3 bands, (c) 4 bands.

Fig. S2 Differences in performance base on the coefficient of determination (R^2) for BPNN for the 13 VZAs.

Fig. S3 Changes in RMSE with more PLSR factors, in the forward and backward scattering directions.

Fig. S4 Loading weights over wavelength for first, second, and third latent variables in the 13 VZAs. (a) PC1, (b) PC2, (c) PC3.

Fig. S5 Differences in performance based on the coefficient of determination (R^2) for PLSR, BPNN, DVI and DDn for the 13 VZAs.

Fig. S6 Simple drawing of the multi-angular reflectance measurements used in this study.

Fig. S7 The flow chart of experimental setup.

Supplementary Table

Table S1 Zenith and azimuth angles of the Sun for all study sites

	Date	Stage	Solar zenith angle (°)	Solar azimuth angle (°)
Exp.1 & 2 (2012)	26th April	Booting	27.74---21.37	133.77---165.91
	5th May	Heading	46.15---41.19	146.40---166.86
	15th May	Initial-filling	42.39---37.22	144.59---166.69
	25th May	Mid-filling	38.58---33.21	142.60---166.64
Exp. 3 & 4 (2013)	26th March	Jointing	38.30---32.91	142.44---166.64
	10th April	Booting	32.84---27.07	138.85---166.58
	25th April	Heading	28.08---21.77	134.22---166.02
	10th May	Initial-filling	24.44---17.37	128.45---164.23
	20th May	Mid-filling	22.78---15.10	124.28---162.01
Exp. 5 & 6 (2014)	20th March	Jointing	40.67---35.41	143.72---166.66
	4th April	Booting	35.05---29.45	130.41---152.54
	26th April	Heading	27.87---21.52	133.95---165.96
	18th May	Mid-filling	23.10---15.56	125.21---162.58
Exp. 7 & 8 (2014)	8th April	Booting	34.12---30.50	138.20---150.39
	23th April	Heading	36.77---31.61	118.83---128.10
	5th May	Initial-filling	36.90---31.28	110.60---118.66
	21th May	Mid-filling	34.64---28.78	105.07---112.54

Supplementary Figure S1

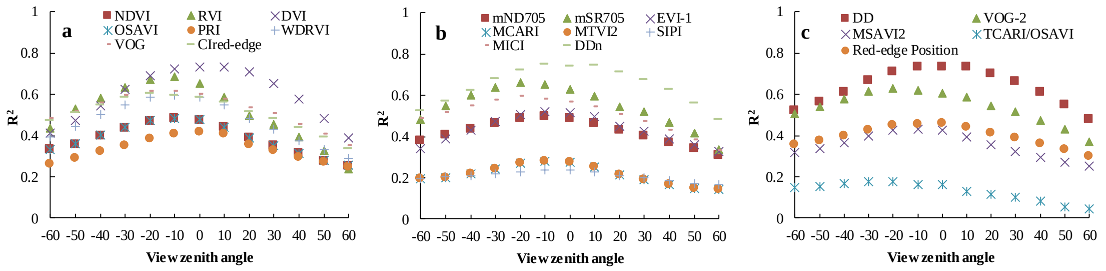

Fig. S1

Supplementary Figure S2

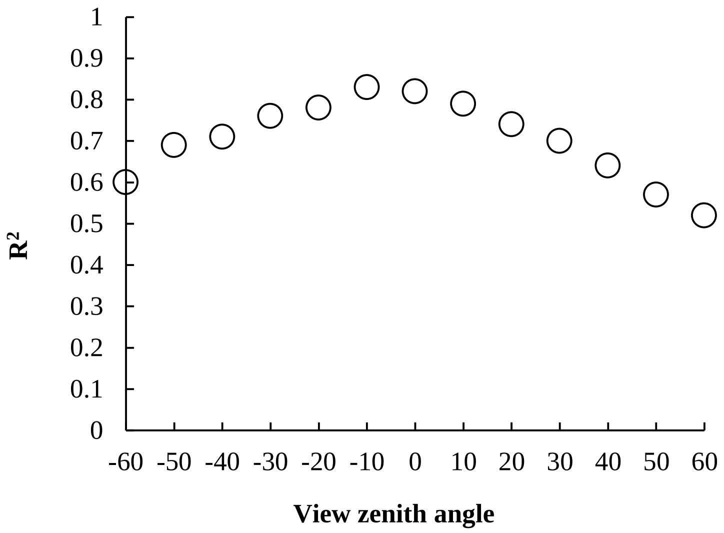

Fig. S2

Supplementary Figure S3

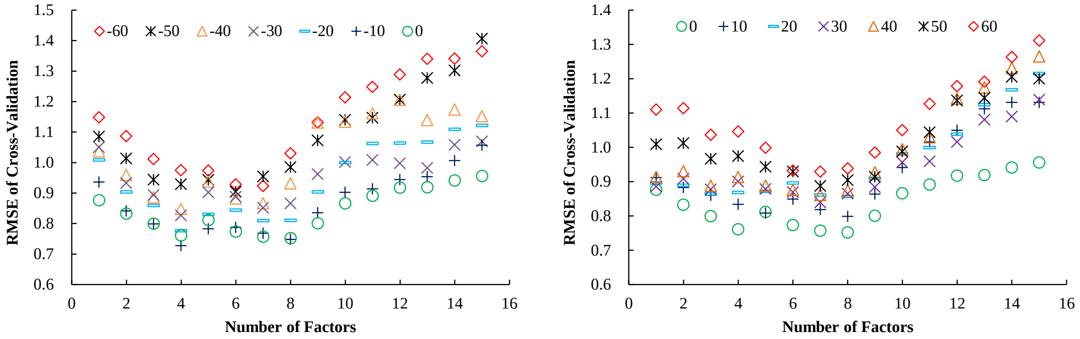

Fig. S3

Supplementary Figure S4

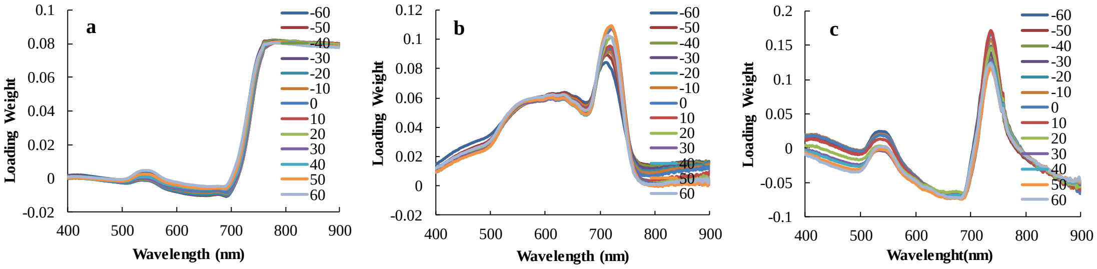

Fig. S4

Supplementary Figure S5

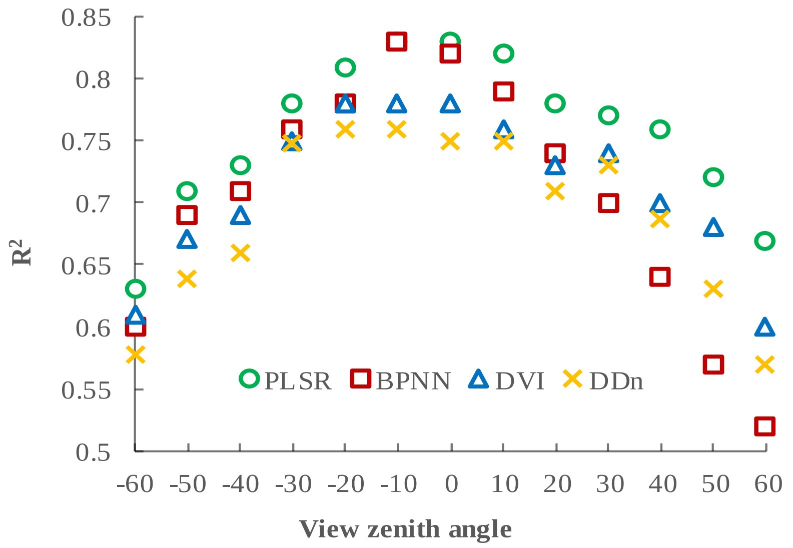

Fig. S5

Supplementary Figure S6

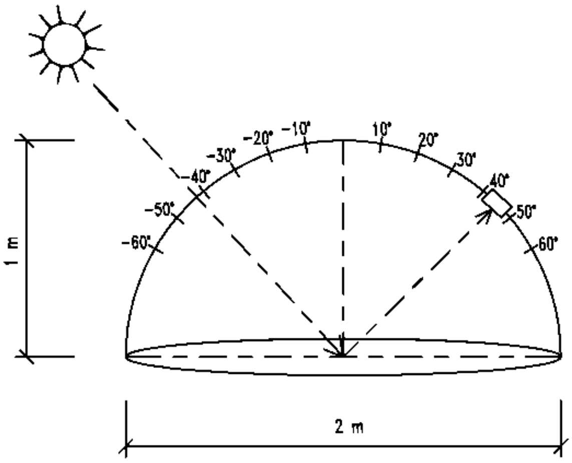

Fig. S6

Supplementary Figure S7

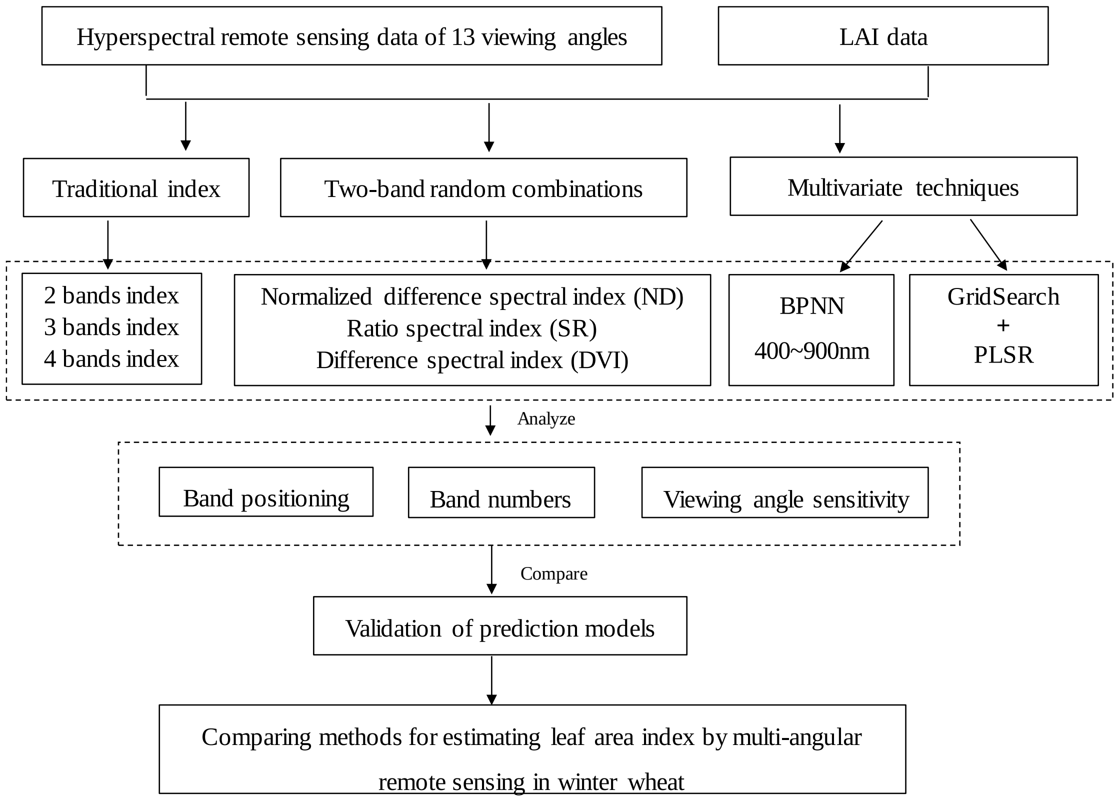

Fig. S7
